# Beyond Bone Remodeling: Denosumab’s Multisystemic Benefits in Musculoskeletal Health, Metabolism, and Age-Related Diseases—A Narrative Review

**DOI:** 10.3390/biomedicines13030732

**Published:** 2025-03-17

**Authors:** Yi-Ting Hung, Wen-Tien Wu, Ru-Ping Lee, Ting-Kuo Yao, Kuang-Ting Yeh

**Affiliations:** 1 School of Medicine, Tzu Chi University, Hualien 970374, Taiwan; 107311156@gms.tcu.edu.tw (Y.-T.H.); timwu@tzuchi.com.tw (W.-T.W.); 2 Department of Orthopedics, Hualien Tzu Chi Hospital, Buddhist Tzu Chi Medical Foundation, Hualien 970473, Taiwan; tayao0318@tzuchi.com.tw; 3 Institute of Medical Sciences, Tzu Chi University, Hualien 970374, Taiwan; fish@gms.tcu.edu.tw; 4 Graduate Institute of Clinical Pharmacy, Tzu Chi University, Hualien 970374, Taiwan; 5 Clinical Education, Hualien Tzu Chi Hospital, Buddhist Tzu Chi Medical Foundation, Hualien 970473, Taiwan

**Keywords:** osteoporosis, sarcopenia, osteosarcopenia denosumab, RANKL inhibitor, RANK/RANKL/OPG pathway (RRO pathway)

## Abstract

**Background:** Denosumab, a receptor activator of nuclear factor kappa-Β ligand (RANKL) inhibitor, demonstrates therapeutic effects beyond traditional osteoporosis management through the RANK/RANKL/osteoprotegerin pathway. **Methods:** This narrative review analyzed 37 studies (2018–2024) examining denosumab’s broader physiological effects and clinical applications. **Results:** Long-term safety data spanning 10 years showed sustained fracture prevention efficacy with a favorable benefit/risk profile. Compared to bisphosphonates, denosumab demonstrated superior outcomes in bone mineral density improvement and fracture risk reduction, particularly in elderly and frail populations. It enhanced muscular function by improving appendicular lean mass and grip strength while reducing fall risk. The drug showed potential cardiovascular benefits through its effects on cardiac and smooth muscle function. Notably, denosumab use was associated with reduced Type II diabetes mellitus risk through improved glucose metabolism. Additionally, it demonstrated promise in osteoarthritis treatment by suppressing osteoclast activity and chondrocyte apoptosis. While there are multisystem benefits, vigilance is required regarding adverse events, including hypocalcemia, infection risk, cutaneous reactions, and osteonecrosis of the jaw. **Conclusions:** Denosumab exhibits potential benefits in bone and systemic metabolism. Further research is needed to fully understand its therapeutic potential beyond osteoporosis and optimize clinical applications across different populations.

## 1. Introduction

The physiological mechanism of bone balance is primarily controlled through the coordination of receptor activator of nuclear factor kappa-Β (RANK), its ligand “RANKL,” and the osteoclastogenesis inhibitor “osteoprotegerin (OPG),” known as the RANK/RANKL/OPG pathway (RRO pathway) [1]. The normal physiological process of bone resorption occurs when RANKL binds to its receptor, RANK, triggering a series of signaling events that promote the differentiation and activation of osteoclasts, leading to bone resorption [1]. Certain substances, such as calcitriol, estrogen, and parathyroid hormone (PTH), regulate the RRO pathway by preventing the interaction between RANK and RANKL, thus inhibiting osteoclast formation and preventing bone loss [2,3]. The RRO pathway not only regulates bone balance in the body but is also involved in the physiological regulation of the muscular system (cardiac, skeletal, and smooth muscles) [4] and the incidence of Type II diabetes mellitus (T2DM) [5,6,7], linking it closely to human health and diseases. Therefore, further studies are required to clarify its physiological mechanisms in the human body.

From an embryological perspective, both muscles and bones originate from the mesoderm and share several common interstitial precursors [8]. Anatomically, skeletal muscles and bones belong to the motor system, with the skeletal muscles attached to the bones, generating movements through contraction. Skeletal muscles have specific endocrine functions, and muscle factors secreted by skeletal muscles can influence bone growth and development [9,10,11]. Similarly, bone factors secreted by bones can also affect muscle strength and mass to some extent [12]. Additionally, various molecular signaling pathways may simultaneously regulate both muscles and bones, whereas local signaling factors can influence their growth and development [13,14]. Skeletal muscles are now known to activate the RRO pathway [15,16,17]. According to recent consensus, the RRO pathway activated through skeletal muscles influences the storage of calcium ions and the activity of the sarcoplasmic/endoplasmic reticulum calcium ATPase, which affects muscle contraction [16]. However, this is merely the tip of the iceberg of all related physiological mechanisms. Further research is needed to clarify how the RRO pathway interacts with cytokines (for example, myokines and osteokines) and proinflammatory factors in skeletal muscle physiology [4].

Denosumab is a widely used drug that targets the RRO pathway in the treatment of osteoporosis. It is a RANKL inhibitor, which blocks the RRO pathway and inhibits osteoclast differentiation and activation that leads to bone resorption. After the Fracture Reduction Evaluation of Denosumab in Osteoporosis Every 6 Months (FREEDOM) study published in 2009, the medicine was approved for clinical use for approximately 15 years [18].

In view of skeletal muscle function, recent research has compared denosumab and bisphosphonates in patients with osteoporosis. They evaluated the effects of both treatments over time on various factors, including appendicular lean mass (ALM), grip strength, fall risk assessment, Time Up and Go (TUG) test performance, and improvements in walking speed, fracture risk, and bone mineral density (BMD), which affect daily functions and quality of life [19,20,21,22]. Overall, denosumab may have considerable potential in patients with both osteoporosis and sarcopenia, as it could enhance daily quality of life by improving muscle strength and BMD [23]. In contrast, in view of cardiac muscle function, the RRO pathway has been associated with cardiovascular mortality, cardiac remodeling, heart failure, and immune or inflammatory cardiomyopathy, which is linked to higher concentrations of RANK [24,25,26,27]. Denosumab reduces RANK levels through its effect on the RRO pathway, potentially leading to a further decrease in the risk of cardiovascular mortality and the incidence of heart failure. Regarding smooth muscle function, RANKL activates vascular smooth muscle myofibroblasts, leading to calcification of arteries and veins, which can result in systemic cardiovascular diseases such as hypertension [28,29,30,31].

Since denosumab has these new extra-beneficial effects for patients with osteoporosis other than osteoporosis treatment, we want to summarize these extra-beneficial effects. In this study, 31 studies on the effects of denosumab in osteoporosis treatment were reviewed. Furthermore, we discussed the relationship between denosumab, T2DM, osteoarthritis, and improvement of skeletal muscle function.

## 2. Materials and Methods

The search strategy for this narrative review was meticulously designed to encompass a comprehensive range of studies examining the effects of denosumab on the muscular system (skeletal, smooth, and cardiac muscles), daily function, quality of life, risk of falls, and incidence of T2DM. A systematic search was conducted using electronic databases including PubMed and Google Scholar, focusing on the literature published between 2018 and 2024. The search methodology employed various combinations of keywords and Medical Subject Heading terms, including “denosumab,” “RANKL inhibitors,” “RRO pathway”, “Osteoporosis”, “Muscle performance”, and “Daily function” to maximize the retrieval of relevant articles.

The inclusion criteria were studies specifically reporting denosumab treatment in patients with osteoporosis published between 2018 and 2024, including case studies, clinical trials, observational studies, and review articles. Studies published before 2018 and ongoing studies without results were excluded from this review. The findings were qualitatively synthesized, focusing on the effects of denosumab treatment on osteoporosis-related outcomes, including muscle performance, quality of life, improvement in daily function, risk of falls, incidence of T2DM, osteoarthritis, and other relevant cardiovascular conditions such as hypertension.

## 3. Results

After a thorough analysis, 37 studies were included in this review and are summarized in Table 1. The key findings of the selected articles are summarized below:

### 3.1. Long-Term Use of Denosumab and Its Related Clinical Outcomes

Denosumab has been approved for clinical use for approximately 15 years since 2009, and many studies have documented its short- and long-term use data and analysis. Ferrari et al. (2019) analyzed FREEDOM and its extension study groups exposed to denosumab for 1–3, 4–7, and 4–10 years for the occurrence of non-vertebral fractures (NVFs). They found that long-term denosumab treatment, for >3 and ≤10 years, was associated with a reduction in NVF incidence compared to that for <3 years (Table 2) [32]. Furthermore, Ferrari et al. (2020) quantitatively assessed the balance between long-term fracture reduction benefits of denosumab treatment and the occurrence of atypical femoral fracture and osteonecrosis of the jaw (ONJ) and demonstrated that long-term denosumab treatment in postmenopausal women with osteoporosis has a favorable skeletal benefit/risk profile for up to 10 years of continuous treatment (Table 2) [33]. Similarly, Kendler et al. analyzed FREEDOM and its extension studies to compare subsequent osteoporotic fracture rates between denosumab- and placebo-treated participants. Their data demonstrated that denosumab decreased the risk of subsequent fracture, and sustained fractures despite denosumab treatment were not necessarily indicative of an inadequate treatment response (Table 2) [34]. Their findings emphasized the advantages and safety of the long-term use of denosumab [32,33,34]. Kendler et al. reviewed the use of denosumab in the treatment of osteoporosis for 10 years. They concluded that denosumab is a potent antiresorptive medication for the treatment of osteoporosis, with clinical trial data for up to 10 years of treatment demonstrating its safety and efficacy in reducing fracture risk (Table 2) [35]. Similarly, Bandeira et al. reviewed the use of denosumab in the treatment of osteoporosis for 10 years. The 10-year experience with denosumab provided data on the efficacy of the drug in terms of safety, reduced fracture incidence, and continued increase in BMD (Table 2) [36]. Di Lorenzo concluded that the long-term use of denosumab for 8 years is both safe and effective (Table 2) [37]. Regardless of the aforementioned long-term benefits of denosumab, we need to consider “denosumab discontinuation”, known as a “drug holiday.” In an analysis by Cummings et al., it was found that denosumab discontinuation causes a temporary spike in bone resorption markers, reaching their highest levels approximately 12 months after the last dose. This is accompanied by a loss of BMD and a fracture risk that returns to that of untreated patients (Table 2) [38]. Therefore, in osteoporosis treatment, lifelong treatment with denosumab should be considered, and if denosumab discontinuation is necessary, a treatment transition from denosumab must be implemented. To mitigate these effects, transitioning to antiresorptive therapy, such as bisphosphonates (e.g., zoledronic acid), is recommended. In Sølling AS et al.’s review, they concluded that short-term denosumab use (≤2.5 years) followed by bisphosphonates can help maintain BMD, but long-term denosumab use (>2.5 years) often requires multiple doses of bisphosphonates to control rebound effects on bone loss (Table 2) [39]. Therefore, regular monitoring of bone turnover markers and BMD is essential to guide post-denosumab treatment decisions.

### 3.2. Denosumab and Its Four Main Adverse Events

The four most common adverse events (AEs) associated with denosumab treatment are hypocalcemia, severe infection, cutaneous adverse reactions, and ONJ.

#### 3.2.1. Hypocalcemia

The FREEDOM and a review reported a 0.05–1.70% incidence of hypocalcemia in denosumab-treated postmenopausal women with osteoporosis [18,40]. The retrospective study by Tsvetov G et al. included 2005 patients (93% women, mean age 76 ± 9 years) (Table 3) [41]. After analysis, hypocalcemia developed during treatment in 149 patients (7.4%): in 66 after 0.5–1, in 48 after 1–2, and in 35 after >2 years. The actual incidence of denosumab-induced hypocalcemia could be higher than that previously reported. Furthermore, the strongest predictors of hypocalcemia were pre-treatment levels of albumin-adjusted serum calcium (*p* < 0.05) and creatinine (*p* < 0.05) (Table 3). Sex, age, 25-hydroxyvitamin-D, PTH, alkaline phosphatase, and denosumab administration as first- or advanced-line osteoporotic therapy had no predictive value (Table 3). Dadana et al. published a case report of denosumab-induced hypocalcemia [42] (Table 3). Bird et al. retrospectively examined the incidence and comparative risk of severe hypocalcemia with denosumab compared to that with oral bisphosphonates among dialysis-dependent patients treated for osteoporosis [43]. They concluded that denosumab was associated with a markedly higher incidence of severe hypocalcemia in female dialysis-dependent patients aged ≥65 years compared to oral bisphosphonates (Table 3). Kunizawa et al. conducted a prospective cohort study to evaluate 383 patients (*n* = 121 hemodialysis/HD; 203 non-hemodialysis/non-HD) for BMD changes and to clarify the safety of denosumab in patients with CKD for 1 year [44]. They found that the incidence of hypocalcemia (defined as Ca < 8.5 mg/dL) was significantly greater in HD than in non-HD patients (*p* < 0.001) (Table 3). Furthermore, in most cases, this decline occurred within 10 days after denosumab injection. Moreover, they found that the occurrence of hypocalcemia and a decrease in serum calcium were associated with higher baseline TRACP-5b (a bone resorption marker) levels, even after adjusting for possible confounders. Therefore, they thought that the baseline TRACP-5b could be a predictor of denosumab-induced hypocalcemia in HD patients (Table 3).

#### 3.2.2. Severe Infections

Diker-Cohen et al. reviewed 33 studies involving 22,253 patients and performed a meta-analysis to evaluate the risk of serious adverse events of infections (SAEI) in denosumab-treated patients [45]. They concluded that a higher incidence of SAEI was observed during treatment with denosumab at an osteoporosis dose (Table 4). However, the overall risk of infection or related mortality was similar in the compared groups (Table 4). Huang et al. conducted a population-based national cohort study with 30,106 pairs of case and control patients from August 2011 to December 2017 to evaluate the infection risk in patients with osteoporosis after long-term denosumab treatment [46]. They concluded that denosumab therapy was associated with a higher risk of infection during the early treatment period (Table 4). However, the risk attenuated significantly after the second year of therapy, and the longer the duration of denosumab treatment, the lower the risk of developing infections (Table 4). Considerable evidence indicates that RANK or RANKL mRNA expression is present in immune tissues besides osteoblasts, osteocytes, and bone stroma (Table 4) [46]. For instance, RANKL expression is essential for T cell function and is believed to enhance T cell activation by acting on dendritic cells (DCs). This interaction not only supports DC survival but also initiates a reciprocal immune response. As a result, the engagement between T cells and DCs promotes the differentiation of T cell subsets into Th1, Th2, and Th17 cells [46]. Although RANKL regulates bone metabolism, it remains uncertain whether inhibiting RANKL could also weaken the systemic immune response (Table 4) [46]. So far, no consistent findings have been reported regarding this issue and its impact on the immune response [46].

#### 3.2.3. Cutaneous Adverse Reactions

Currently, the whole mechanisms about how denosumab causes cutaneous adverse reactions still remain unknown, and further studies are needed to clarify and discuss the mechanisms. There are two case reports, reported by King et al. and Al-Attar et al. [47,48], that found that denosumab may be associated with cutaneous adverse reactions, informing clinicians of this phenomenon (Table 5).

#### 3.2.4. ONJ

Ferrari et al. (2020) reported their analysis of ONJ (Table 6) [33]. The exposure-adjusted subject incidence of ONJ in the long-term group was 35 per 100,000 subject-years, corresponding to 7 observed cases. Among these seven cases, there were two mild and five moderate with no severe or life-threatening cases. Furthermore, six of these cases were resolved, and one was ongoing at the end of their study. The skeletal benefit/risk ratio (fractures prevented per adverse event observed) of denosumab for clinical fractures was 40 for ONJ. Considering the number of fractures prevented in relation to the severity of observed AEs, the benefit/risk ratio of denosumab is even more favorable than the previously stated 40 clinical fractures prevented per ONJ case. Watts et al. analyzed the FREEDOM and its extension studies for effects of invasive oral procedures and events (OPEs) (for example, dental implants, tooth extraction, natural tooth loss, scaling/root planning, and jaw surgery) and presented the details of positively adjudicated ONJ cases during 7 years in denosumab-treated women [49]. They found that although invasive OPEs were common in denosumab-treated women and associated with an increased ONJ incidence, the overall incidence of ONJ was low, and all cases with a complete follow-up resolved with treatment (Table 6). Beth-Tasdogan et al. conducted a review and concluded that treatment objectives for patients with a definite diagnosis of medication-related ONJ are to control the infection of soft and hard tissues and minimize the progression or occurrence of bone necrosis to optimize wound healing (Table 6) [50].

### 3.3. Denosumab Versus Bisphosphonates in the Treatment of Osteoporosis

Denosumab and bisphosphonates are two widely used drugs for the treatment of osteoporosis worldwide. Several studies have compared the safety, efficacy, and clinical outcomes of these drugs. Lyu et al. identified 10 eligible randomized controlled trials for meta-analysis [51]. They concluded that denosumab resulted in a significantly greater improvement in BMD than bisphosphonates in the lumbar spine, total hip, and femoral neck at 12 and 24 months and that only one study demonstrated a greater reduction in the occurrence of osteoporotic fracture with denosumab treatment (Table 7). Brown et al. conducted a retrospective cohort study focusing on mortality in older adults following fragility fractures [52]. They demonstrated that denosumab may provide better outcomes than bisphosphonates in terms of improving bone density and reducing fracture risk, even in frail and elderly individuals (Table 7). Kim et al. conducted a retrospective observational cohort study focusing on denosumab versus zoledronic acid in elderly patients after hip fractures [53]. They found that, in frail and elderly populations, half-yearly denosumab treatment was superior to yearly zoledronic acid treatment in terms of BMD improvement and demonstrated a significantly higher persistence rate (Table 7). Kobayashi et al. identified 25 eligible randomized controlled trials for a meta-analysis [54]. They concluded that, compared to bisphosphonates, denosumab was significantly associated with fewer rates of withdrawal due to AEs and vertebral fractures, making it more suitable for elderly patients with osteoporosis (Table 7). Although denosumab shows better outcomes than bisphosphonates, we need to consider their difference in “discontinuation.” Unlike bisphosphonates, which can continue to provide pharmacological effects that promote bone quality for many years after discontinuation, denosumab rapidly loses its bone-quality-enhancing effects upon discontinuation and is associated with rebound effects, including rapid BMD loss and increased fracture risk, particularly vertebral fracture, due to rapid increase in bone turnover (Table 7) [39].

### 3.4. Denosumab and Improvement of Daily Function, Muscle Performance, Quality of Life, Bone Quality, Bone Microarchitecture, and Bone Turnover Biomarkers

Bonnet et al. compared the effects of denosumab in postmenopausal women treated for osteoporosis for an average duration of 3 years and similar women without treatment or who received bisphosphonates (alendronate or zoledronate) [19]. Both denosumab and bisphosphonates improved BMD compared to the untreated group (Table 8). However, only denosumab increased the ALM and handgrip strength (Table 8). Furthermore, changes in ALM and handgrip strength were strongly correlated with changes in lumbar spine BMD in the denosumab group but not in the other groups (Table 8). Miedany et al. analyzed three groups: denosumab/zoledronate/alendronate at 5, 3, and 5 years of treatment [20]. Their results showed that, compared to baseline, there was a significant increase in the BMD at both the spine and hip and vitamin D levels and a significant decrease in the fracture risk score in all three groups. The denosumab group showed a highly significant decrease in the fall risk score and significant improvements in grip strength, TUG test performance, and gait speed (Table 8). In the zoledronate and alendronate groups, the results showed significant improvement in TUG test performance and gait speed; however, there was no significant change in fall risk (Table 8). Rupp et al. analyzed 120 outpatients with osteoporosis who were categorized into three groups: basic vitamin D treatment, denosumab, and bisphosphonate [21]. After a mean follow-up period of 17.6 ± 9.0 months, they found a significantly higher increase in grip strength in both the denosumab and bisphosphonate groups compared to that in the vitamin D group. Furthermore, the denosumab group showed a significantly greater increase in chair rising test force than the bisphosphonate group (Table 8). Changes in BMD and bone metabolic parameters were not associated with changes in muscle performance. Elderly patients receiving denosumab treatment showed a stable muscle mass, indicating a protective effect against sarcopenia. Compared to bisphosphonates, denosumab resulted in increased muscle strength in both the upper and lower limbs, indicating its systemic rather than localized effects. Phu et al. compared the effects of denosumab and zoledronate on muscle strength, balance, and function in elderly individuals at risk of falls and fractures who were treated with vitamin D supplementation for 6 months [22]. The denosumab and zoledronate groups included 51 and 28 patients, respectively. The results showed that denosumab improved walking speed and TUG test and Four-Square Step Test (FSST) performances and reduced the fear of falling, with a trend toward improvement in Short Physical Performance Battery scores and stability limits (Table 8). Zoledronic acid also improved walking speed and TUG test performance; however, the effects were less pronounced than those of denosumab (Table 8). There were no significant differences between the two groups in terms of falls or fractures. This study highlights the potential of denosumab in improving muscle performance and reducing fall risk in older adults, suggesting that it may be superior to zoledronic acid for certain physical functions. Kawakami et al., Davenport et al., and Rochette et al. reported the same conclusion that the RRO pathway might regulate vascular smooth muscles and lead to vascular and cardiac valvular calcification [28,29,30,31].

As for bone quality and microarchitecture, although BMD is the most commonly used measure for assessing bone health and fracture risk, many individuals who experience fractures have osteopenia or even normal BMD (Table 8) [36]. This highlights the importance of other factors, such as bone microarchitecture, in determining bone quality. A transiliac bone biopsy can provide a direct evaluation of bone microarchitecture, but it is an invasive procedure and not widely used in clinical practice. Bone biopsy results show that denosumab is linked to normal bone histology, a very low remodeling rate, and increased mineralization density. Furthermore, high-resolution peripheral quantitative computed tomography (HR-pQCT) has provided valuable insights into bone microstructure. This imaging technique can assess volumetric bone density as well as cortical and trabecular bone microarchitecture. While bone biopsy and HR-pQCT provide highly detailed assessments, they are not widely available in clinical practice. A more accessible way and alternative is the trabecular bone score (TBS), a software-based enhancement of lumbar spine dual-energy X-ray absorptiometry (DXA). TBS analyzes the texture of the lumbar spine DXA image to estimate bone microarchitecture. Shevroja E et al. reviewed 96 articles and then concluded the following: (1) TBS can predict fragility fractures independently of BMD and other clinical risk factors; (2) TBS is useful for individuals whose fracture risk assessment tool (FRAX) score or BMD T-score is near the treatment intervention threshold; (3) TBS, when used alongside BMD, is a valuable tool for assessing an individual’s response to long-term denosumab treatment, especially over a period of 5 years or more; (4) TBS can be used alongside BMD to provide information on bone microarchitecture; and (5) since TBS reflects aspects of bone microarchitecture, a low (degraded or partially degraded) TBS may suggest the need for treatments (Table 8) [55].

Bone turnover biomarkers (BTBs) are categorized as “Bone Formation Markers” and “Bone Resorption Markers.”

(1) Bone Formation Markers:
  Bone-specific alkaline phosphatase (BALP), Procollagen type I N-propeptide (PINP), and Osteocalcin (OC).
(2) Bone Resorption Markers:
  C-telopeptide cross-linked type I collagen (β-CTX), Urine N-telopeptide cross-linked type I collagen (NTX), and Tartrate-resistant acid phosphatase 5b (TRACP-5b).
  In view of their clinical use, Brown et al. concluded nine points as follows (Table 8) [56]:

(1) Diagnosis of Postmenopausal Osteoporosis:
  Osteoporosis is identified using BMD testing or the occurrence of a low-energy fracture. Although BTBs are often elevated in osteoporosis, they are not used for diagnosis but may help detect underlying conditions.
(2) Prediction of Bone Loss in Untreated Postmenopausal Women:
  BTBs tend to rise during menopause due to increased bone turnover caused by estrogen deficiency. While there is a general link between high BTBs and bone loss, they are not reliable for predicting individual cases.
(3) Prediction of Fractures in Untreated Postmenopausal Women:
  BTBs are not included in standard fracture risk assessment tools like FRAX due to insufficient supporting data.
(4) Selection of Pharmacological Treatment:
  Theoretically, high BTBs suggest antiresorptive therapy might be more beneficial, while low BTBs indicate a potential advantage from anabolic treatment. However, BTBs are not currently a primary factor in treatment selection.
(5) Monitoring of Response to Therapy in Postmenopausal Osteoporosis:
  Since BTBs change quickly after starting treatment, they can be used to assess early response to therapy. However, their role in predicting long-term fracture risk remains uncertain.
(6) Use of Bone Turnover Markers for Optimizing Adherence to Therapy:
  Many patients discontinue osteoporosis treatment over time. While BTBs could be used to check adherence, their effectiveness in improving long-term compliance is unclear.
(7) Managing Drug Holidays:
  Bisphosphonates continue to have an effect even after stopping treatment, allowing for temporary breaks. BTBs can be used to monitor when therapy should resume, but they do not accurately predict future fracture risk.
(8) Managing Cessation of Denosumab Therapy:
  Denosumab discontinuation leads to a sharp increase in bone resorption, raising the risk of fractures. Switching to bisphosphonate may help reduce this risk.
(9) Risk Assessment for Atypical Femoral Fractures (AFFs) and ONJ:
  Long-term use of strong antiresorptive drugs can, in rare cases, lead to AFF and ONJ. Although BTBs may help assess changes in bone metabolism, they are not yet proven to predict these conditions.

### 3.5. Denosumab and T2DM Incidences

Recently, the correlation between RANKL inhibition and improved glucose metabolism or promotion of beta-cell proliferation has been discussed (Table 9) [5,6,57]. Lyu et al. compared the effects of denosumab and oral bisphosphonates in reducing the risk of T2DM in adults with osteoporosis [57]. After analysis, they concluded that denosumab use was associated with a lower risk of incident T2DM than oral bisphosphonate use in adults with osteoporosis (Table 9). Huang et al. conducted a nationwide propensity score-matched cohort study to evaluate whether denosumab use was associated with a lower risk of developing T2DM in patients with osteoporosis [5]. They concluded that denosumab treatment was associated with a lower risk of incident diabetes, and several sensitivity analyses also demonstrated similar results of a lower diabetes risk associated with denosumab treatment (Table 9). Henney et al. performed a retrospective propensity score-matched analysis to evaluate the impact of denosumab on (1) the incidence of T2DM and (2) long-term health outcomes (microvascular [neuropathy, retinopathy, and nephropathy] and macrovascular [cardiovascular disease and cerebrovascular accident] complications, and all-cause mortality) in patients with T2DM [6]. They observed that denosumab treatment was associated with a reduced risk ratio (RR) for incident T2DM as well as an associated reduced RR for all-cause mortality and microvascular complications (Table 9). The aforementioned studies may help physicians choose an appropriate anti-osteoporosis medication for patients with osteoporosis while considering the risk of T2DM. More importantly, further studies are needed to clarify the entire mechanism of the RRO pathway and the related physiological mechanisms associated with T2DM (Table 9).

### 3.6. Denosumab and Osteoarthritis

Herrero-Beaumont et al. discussed that denosumab might affect the subchondral bone (SB) quality of patients with osteoarthritis (OA) when acting as a bone former (Table 10) [58]. However, the characteristics of this newly formed bone are unclear as its formation is not paralleled by an increase in serum anabolic biomarkers. Furthermore, how the phenomenon affects the SB quality of patients with OA in the long term still remains unknown. Shangguan et al. conducted several tests to assess the effects of denosumab on osteoclast activity and chondrocyte apoptosis and to assess the impact of denosumab on the NF-κB pathway [59]. Furthermore, they used an OA model to explore the influence of denosumab on subchondral bone remodeling and cartilage degeneration in vivo. Their findings indicate that denosumab could suppress osteoclast activity and chondrocyte apoptosis, thereby mitigating OA-related subchondral bone remodeling and cartilage degeneration (Table 10). According to the findings, they provide a mechanistic basis for OA treatment with denosumab. Yu et al. conducted a population-based cohort study to evaluate the incidence of total knee arthroplasty (TKA), a marker of severe knee OA, among older females with concurrent knee OA and osteoporosis who were treated with denosumab or bisphosphonates [60]. By analysis of a total of 336 patients underwent TKA [*n* = 121 (1.8%)] in the denosumab group and [*n* = 215 (3.1%)] in the bisphosphonate group during the follow-up period, it was found that those treated with denosumab had a lower TKA incidence than those treated with bisphosphonates (6.9 vs. 8.5 per 1000 person-years). The adjusted hazard ratio (aHR) for TKA in the denosumab group was 0.77 (*p* = 0.024), indicating a 23% reduction in the risk of severe knee OA requiring surgical intervention compared with the bisphosphonate group. These results support the hypothesis that denosumab might benefit subchondral health, potentially slowing OA progression and delaying TKA (Table 10). Wittoek et al. conducted an RCT with denosumab to evaluate the effects on structure modification in erosive hand OA [61]. They focused on the change in the total Ghent University Scoring System (GUSS) in the 24th week, where positive changes correspond to remodeling and negative changes to erosive progression. After analysis, the GUSS results had statistically significant differences in both the 24th week and the 48th week. Furthermore, markedly less new erosive joints developed through the 48th week and improvement in pain and disability through the 96th week in the denosumab group. They provided a concept that structural damage in erosive hand OA might be modulated by denosumab (Table 10).

## 4. Discussion

**Figure 1:**
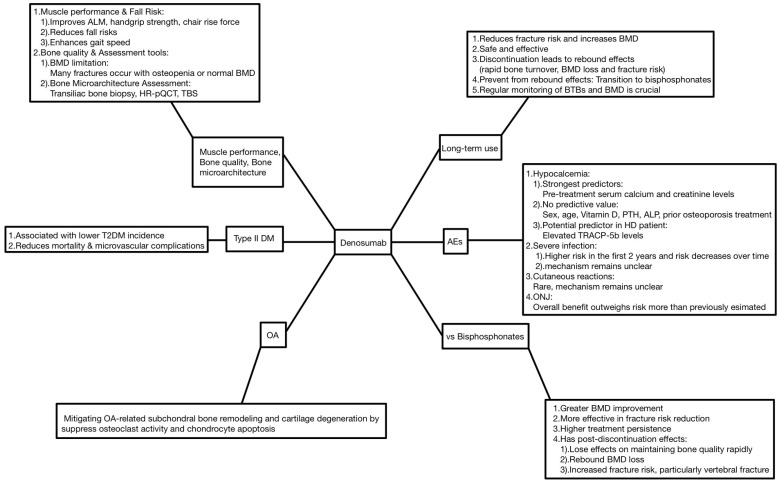
Key summary points. (BMD: bone mineral density; BTBs: bone turnover biomarkers; AEs: adverse events; PTH: parathyroid hormone; ALP: alkaline phosphatase; ONJ: osteonecrosis of the jaw; ALM: appendicular lean mass; TBS: trabecular bone score; OA: osteoarthritis).

Denosumab has been in use clinically for approximately 15 years since 2009. Our review, encompassing a range of studies, provides a multifaceted understanding of denosumab, highlighting the complexity of long-term use and common AEs, compared to bisphosphonates, impact on muscle performance, and Type II DM incidence (Figure 1).

### 4.1. Longitudinal Assessment of Denosumab Treatment Outcomes

The aforementioned reports [32,33,34,35,36,37] concluded that the long-term use of denosumab is advantageous in reducing the incidence of fractures, particularly the NVF, and in continuing to increase BMD in long-term use. Furthermore, all of their findings emphasized the safety of the long-term (>3 years) use of denosumab. Regarding the long-term use of denosumab, there is sufficient proof of its safety, reduced fracture incidence, and continued increase in BMD.

Although there are many long-term benefits from denosumab, we still need to take “denosumab discontinuation” into consideration. Due to its rebound effect on the temporary spike of bone resorption markers accompanied by loss of BMD and higher fracture risk, life-long treatment with denosumab in osteoporosis should be considered [38]. However, if denosumab discontinuation is necessary, a treatment transition to antiresorptive therapy, such as bisphosphonates, must be implemented. Therefore, regular monitoring of bone turnover biomarkers and BMD is essential to help post-denosumab treatment decisions.

### 4.2. Denosumab and Its Common AEs

Common AEs associated with denosumab have several significant aspects. Hypocalcemia is a notable complication primarily attributed to two mechanisms: inhibition of osteoclast activity, which leads to decreased bone resorption and reduced calcium release, and effect on renal calcitriol synthesis. Furthermore, the pre-treatment levels of albumin-adjusted serum calcium and creatinine are the strongest predictors in denosumab-related hypocalcemia, but sex, age, vitamin D, PTH, alkaline phosphatase, or prior osteoporosis treatment do not have predictive value [41]. Given the prolonged duration of action of denosumab of up to 6 months, close monitoring and surveillance to develop appropriate treatment strategies must be needed [42,43]. Moreover, the higher incidence in HD patients compared with non-HD patients was reported, and it was suggested that TRACP-5b could be a predictor in HD patients [44].

Severe infection is another crucial consideration linked to RANK/RANKL expression in immune tissues. A higher incidence of SAEI in the initial 2 years of denosumab treatment at osteoporosis doses was reported, and this risk was significantly attenuated after the second year [45,46]. Regarding cutaneous adverse reactions, two papers documented and confirmed these reactions through skin biopsies, histopathological examinations, and related tests [47,48]. As the complete biophysiological mechanisms of severe infection and cutaneous adverse reactions remain unclear, further studies are needed for clarification. What is even more important is that we as clinicians should keep in mind severe infection and cutaneous adverse reactions when prescribing denosumab for patients undergoing osteoporosis treatment.

Concerning medication-related ONJ, while invasive oral procedures are commonly performed in denosumab-treated women and are associated with an increased ONJ incidence, the overall rate remains low, with all follow-up-completed cases resolving with treatment [49]. Furthermore, we need to keep in mind that the management of patients with osteoporosis receiving denosumab should include routine dental care and treatment.

### 4.3. Clinical Comparison Between Denosumab and Bisphosphonate Interventions for Osteoporosis

As for the choice of medicine in osteoporosis treatment, there is considerable evidence that denosumab is a better choice than bisphosphonates in the treatment of osteoporosis when the patients have poorer BMD and might have a higher fracture rate, especially in frail elderly patients with osteoporosis [51,52,53].

Although denosumab shows better outcomes than bisphosphonates, we need to consider their difference in “discontinuation” before using them in osteoporosis treatment. Bisphosphonates could continue providing effects that promote bone quality for many years after discontinuation, and it does not have rebound effects. However, denosumab could rapidly lose its bone-quality-enhancing effects upon discontinuation, and it is associated with rebound effects with rapid BMD loss and a higher fracture risk due to the rapid increase in bone turnover [39].

### 4.4. Denosumab and Improvement of Daily Function, Muscle Performance, Quality of Life, Bone Quality and Bone Microarchitecture

As reported in the four aforementioned studies [19,20,21,22], denosumab may improve daily function and muscle performance, which are imperative in daily life. Moreover, four studies indicated that the RRO pathway may be associated with cardiovascular mortality, cardiac remodeling, heart failure, and immune or inflammatory cardiomyopathy, linked to higher concentrations of RANK [23,24,25,26]. Furthermore, four studies have indicated that RANKL may activate vascular smooth muscle myofibroblasts, leading to further calcification of blood vessels, which can result in systemic diseases related to vascular issues [27,28,29,30]. Therefore, we provided insight into the use of denosumab in the treatment or cooperative treatment of hypertension or other cardiovascular diseases in the future, owing to its effects on cardiac and smooth muscles. If it works, denosumab is of much more benefit than osteoporosis treatment.

Furthermore, although BMD is the most commonly used measure for evaluating bone health and fracture risk, we need to consider bone microarchitecture in determining bone quality [36]. Currently, there are three ways to evaluate bone microarchitecture: (1) transiliac bone biopsy, (2) HR-pQCT, and (3) TBS. Bone biopsy and HR-pQCT both have restrictions and are not commonly used in clinical practice. As for TBS, it is more widely used in clinical practice.

In view of clinical use of bone turnover biomarkers (BTBs) [56], although they have many restrictions, they can provide much information to help us evaluate or monitor clinical conditions.

### 4.5. Denosumab and T2DM Incidence

Denosumab was associated with a lower risk of incident T2D [5,6,57]. Furthermore, denosumab may be associated with a reduced RR for all-cause mortality and microvascular complications. Recently, an increasing number of studies have investigated the relationship between denosumab and T2D, such as the study by Wang et al. [27]. There are two hypotheses regarding the relationship between denosumab and the lower risk of diabetes: (1) Low-grade inflammation is associated with the onset of insulin resistance and DM. RANKL is a proinflammatory factor that modulates inflammation. Systemic insulin resistance results from the subacute inflammation caused by RANK. Therefore, mitigating RANKL using denosumab may reduce subacute inflammation and improve insulin resistance [7]. (2) RANKL suppression may promote beta-cell proliferation. Beta-cell failure is an essential pathogenic process in DM. The RRO pathway inhibits beta-cell replication in the human body; therefore, suppressing the RRO pathway via denosumab might enhance beta-cell replication in the human body [62,63]. In 2023, Wang et al. designed a 12-month multicenter, randomized controlled trial involving postmenopausal women diagnosed with both osteoporosis and prediabetes to provide evidence on the efficacy of denosumab in modulating glucose metabolism in these patients [27]. Although the study is now under analysis, the results derived from this clinical trial may provide insights into the potential of denosumab in preventing T2DM in high-risk populations and broaden the clinical use of denosumab. Although the physiological mechanisms of denosumab in the RRO pathway in the human body are still not fully understood, denosumab could provide greater benefits than osteoporosis treatment through the RRO pathway. Nonetheless, more studies are needed to clarify the mechanisms underlying the relationship between denosumab and a lower risk of diabetes and the improvement in daily function, muscle performance (skeletal, smooth, and cardiac muscles), and quality of life.

### 4.6. Denosumab and OA: Exploring Dual Benefits in Bone Health and Joint Preservation

Herrero-Beaumont et al. provided a perspective that denosumab might affect the SB quality of patients with OA [58]. Shangguan et al. conducted several tests and used a model to try to clarify and explain the possible mechanism of denosumab in mitigating OA-related SB remodeling and cartilage degeneration [59]. Furthermore, results from two studies support the hypothesis that denosumab might benefit subchondral health, potentially slowing OA progression and acting as a “joint protecter” [60,61]. Based on their findings, they provided a possible clinical use for OA treatment with denosumab.

This study had several limitations. First, our literature search was confined to studies published between 2018 and 2024, potentially overlooking significant findings and valuable perspectives on denosumab in the treatment of osteoporosis from earlier publications. Second, while current osteoporosis treatments encompass five major therapeutic options—bisphosphonates, selective estrogen receptor modulators, PTH analogs, RANKL inhibitors, and hormone replacement therapy—our review primarily focused on the two most commonly prescribed medications: RANKL inhibitors and bisphosphonates. Third, this review overly relies on many observational studies. This selective focus may have limited our understanding of the comprehensive effects of other therapeutic options in humans.

Despite these limitations, our findings provide valuable guidance for clinical decision-making regarding denosumab therapy. The results suggest that several patient populations may particularly benefit from denosumab treatment: patients requiring long-term osteoporosis management (minimum 3 years) because of its demonstrated efficacy in reducing fractures and increasing BMD; frail elderly patients or those with poor BMD and higher fracture incidences who may respond better to denosumab than bisphosphonates; individuals seeking improvements in functional capacity, as evidenced by enhanced handgrip strength, walking speed, and reduced fall rates compared to bisphosphonate therapy; patients at an elevated risk for T2DM, given the potential role of denosumab in reducing T2DM incidence through inflammation mitigation and improved insulin sensitivity; and patients with OA, given the possible mechanism of denosumab in suppressing osteoclast activity and chondrocyte apoptosis. Conversely, certain patient populations warrant careful consideration or may be unsuitable for denosumab therapy: those at risk for hypocalcemia or with kidney dysfunction require vigilant monitoring of serum calcium and creatinine levels; immunocompromised patients or those prone to infections, particularly during the initial two years of treatment when the infection risk is greater; individuals with pre-existing skin conditions or susceptibility to cutaneous reactions; and patients with oral health issues or an increased risk for ONJ. These insights emphasize the importance of personalized medical approaches in osteoporosis management, in which careful patient selection and monitoring are crucial for optimizing treatment outcomes and minimizing AEs.

## 5. Conclusions

Our comprehensive review demonstrates that the role of denosumab, a RANKL inhibitor that targets the RRO pathway, extends significantly beyond its primary role in osteoporosis treatment. Evidence has revealed therapeutic benefits in muscle performance through increased ALM and enhanced handgrip strength while also showing associations with reduced T2DM risk through potential mechanisms of inflammation mitigation and improved insulin sensitivity. Furthermore, emerging evidence suggests its potential effects on cardiac muscle function, vascular smooth muscle regulation, and subchondral bone quality. Based on our findings, denosumab appears to be particularly suitable for several patient populations, including those requiring long-term osteoporosis management (≥3 years), frail elderly patients with poor BMD and high fracture risk, individuals needing improvements in muscle performance, patients with an elevated risk for T2DM, and patients with OA. However, careful consideration is needed for patients with hypocalcemia risk, renal dysfunction, immunocompromised status, or an increased risk of ONJ. Future research should focus on further elucidating the RRO pathway mechanisms in muscle function and metabolism while investigating potential therapeutic applications in cardiovascular diseases. This expanded understanding suggests the potential for broader therapeutic applications beyond osteoporosis and emphasizes the importance of personalized patient selection and monitoring for optimal outcomes.

## Figures and Tables

**Table 1:** Key summary points.

Section	Key Findings
Long-term Use of Denosumab	Long-term use (>3 to ≤10 years) reduces fracture risk and increases BMD. Safe and effective for osteoporosis, with proven benefits up to 10 years. Discontinuation leads to rapid bone turnover, BMD loss, and fracture risk. Transition to bisphosphonates is essential to prevent rebound effects. Regular monitoring of BMD and bone turnover markers is crucial when using denosumab.
Common Adverse Events	Hypocalcemia: (1) Strongest predictors: Pre-treatment serum calcium and creatinine levels. (2) No predictive value: Sex, age, vitamin D, PTH, alkaline phosphatase, or prior osteoporosis treatment. (3) Hypocalcemia can persist for up to 6 months after treatment. (4) Hemodialysis (HD) patients: Higher hypocalcemia incidence. (5) Potential predictor in HD patients: Elevated TRACP-5b levels. Severe Infection: Higher risk in the first 2 years and risk decreases over time. Cutaneous Reactions: Rare, and the mechanism remains unclear. ONJ: (1) Skeletal Benefit/Risk Ratio: Overall benefit outweighs risk more than previously estimated. (2) Findings on ONJ: OPEs were common and linked to increased ONJ risk, but incidence remained low. (3) ONJ Case Outcomes: Almost all cases with complete follow-up resolved with treatment.
Denosumab vs. Bisphosphonates	BMD Improvement: Denosumab showed greater gains in the lumbar spine, total hip, and femoral neck at 12 and 24 months than bisphosphonates. Fracture Risk Reduction: Denosumab is more effective than bisphosphonates, including in frail and elderly patients. Higher Treatment Persistence: Denosumab has lower withdrawal rates and fewer vertebral fractures than bisphosphonates. Post-Discontinuation Effects: (1) Bisphosphonates maintain bone quality for years, but denosumab loses its effects rapidly. (2) Denosumab discontinuation causes rebound BMD loss and increased fracture risk, especially vertebral fractures.
Muscle Performance, Bone Quality, Bone Microarchitecture, and Bone Turnover Biomarkers	Muscle Performance and Fall Risk: Denosumab improves muscle performance (ALM, handgrip strength, chair rise force), reduces fall risk, and enhances gait speed. Bone Quality and Assessment Tools: (1) BMD Limitations: Many fractures occur with osteopenia or normal BMD. (2) Bone Microarchitecture Assessment: A. Transiliac Bone Biopsy: Direct but invasive. B. HR-pQCT: Provides detailed bone structure analysis but is not widely available. C. TBS: Enhances DXA scans, predicts fracture risk independently of BMD, and aids long-term treatment assessment. Bone Turnover Biomarkers (BTBs) in Clinical Use: (1) Diagnosis and Prediction: BTBs aid in detecting osteoporosis but are not diagnostic or predictive of individual fracture risk. (2) Treatment Selection and Monitoring: A. High BTBs suggest antiresorptive therapy, while low BTBs may indicate anabolic treatment. B. BTBs monitor early treatment response but do not predict long-term fracture risk. (3) Therapy Management: A. BTBs help track bisphosphonate drug holidays which are less useful for denosumab discontinuation. B. Denosumab discontinuation increases fracture risk; switching to bisphosphonates may help. C. BTBs may reflect bone metabolism changes but do not predict AFF or ONJ.
Denosumab and Type II DM	Associated with lower Type II diabetes incidence and reduces mortality/microvascular complications. Hypotheses: Reduces inflammation linked to insulin resistance. Promotes beta-cell proliferation by suppressing the RRO pathway.
Denosumab and Osteoarthritis	Denosumab could suppress osteoclast activity and chondrocyte apoptosis, thereby mitigating OA-related subchondral bone remodeling and cartilage degeneration, potentially slowing OA progression and as a treatment method for OA.

**Table 2:** Summary of long-term use of denosumab and its related clinical outcomes.

Author	Research	Results/Conclusion
Ferrari et al. (2019) [32]	RCT	Long-term denosumab treatment (>3 to ≤10 years) reduced non-vertebral fracture incidence compared to <3 years.
Ferrari et al. (2020) [33]	RCT	Denosumab provides a favorable skeletal benefit/risk profile for up to 10 years in postmenopausal osteoporosis patients.
Kendler et al. (2019) [34]	RCT	Denosumab reduces subsequent fracture risk. Sustained fractures do not necessarily indicate an inadequate treatment response.
Kendler et al. (2022) [35]	Review	Denosumab is a potent and safe antiresorptive treatment for osteoporosis. Clinical trial data for up to 10 years confirm its efficacy in fracture risk reduction.
Bandeira et al. (2022) [36]	Review	Continued BMD increases, fracture risk reduction, and safety over long-term use (10 years) of denosumab.
Di Lorenzo (2023) [37]	Review	Denosumab is safe and effective for up to 8 years.
Cummings et al. (2018) [38]	Post hoc analysis of RCT	Denosumab discontinuation causes a rapid spike in bone resorption markers within 12 months, leading to BMD loss and an increased fracture risk, particularly vertebral fracture, due to rapid increase in bone turnover.
Sølling (2022) [39]	Review	Rebound effects of denosumab discontinuation: Discontinuation leads to rapid bone turnover increase, requiring careful management. Strategies for treatment transition from denosumab to other anti-osteoporotic medication: (1) Short-term denosumab use (≤2.5 years): Followed by bisphosphonates can help maintain bone mineral density. (2) Long-term denosumab use (>2.5 years): Often requires multiple doses of bisphosphonates to control rebound bone loss. 3. Post-denosumab treatment strategy: Regular monitoring of bone turnover markers and bone mineral density is essential, and it helps to guide treatment decisions to prevent bone loss and fractures.

**Table 3:** Summary of hypocalcemia.

Author	Research	Results/Conclusion
Tsvetov et al. (2020) [41]	Retrospective cohort study	The rates of hypocalcemia are higher than previously reported 0.05–1.70%. Strongest predictors of hypocalcemia: Pre-treatment levels of albumin-adjusted serum calcium and creatinine. No predictive value: Sex, age, vitamin D, PTH, alkaline phosphatase, or prior osteoporosis treatment.
Dadana et al. (2023) [42]	Case report	The prolonged effect (up to 6 months) of denosumab can cause persistent hypocalcemia beyond the treatment course.
Bird et al. (2024) [43]	Retrospective cohort study	Denosumab had a significantly higher risk of severe hypocalcemia compared to oral bisphosphonates in female dialysis-dependent patients aged ≥65 years.
Kunizawa et al. (2020) [44]	Prospective cohort study	Higher incidence of hypocalcemia in hemodialysis (HD) patients compared to non-HD patients (*p* < 0.001). Higher baseline TRACP-5b levels were associated with a greater risk of hypocalcemia and the suggestion that TRACP-5b could be a predictor of hypocalcemia in HD patients.

**Table 4:** Summary of severe infection.

Author	Research	Results/Conclusion
Diker-Cohen et al. (2020) [45]	Review + meta-analysis	Denosumab at an osteoporosis treatment dose is associated with a higher incidence of serious infection-related events. The overall risk for any infection or related mortality is similar to compared groups.
Huang et al. (2023) [46]	Population-based national cohort study	Denosumab treatment carries a higher infection risk at the early periods of treatment. This risk significantly decreases after the second year of therapy. Longer treatment duration is associated with a lower infection risk. RANK or RANKL mRNA expression is present in immune tissues, but it still remains uncertain whether inhibiting RANKL could also weaken the systemic immune response.

**Table 5:** Summary of cutaneous adverse reactions.

Author	Research	Results/Conclusion
King et al. (2018) [47]	Case report	A 72-year-old woman developed an acute pruritic skin rash 5 months ago. She had been treated with denosumab for osteoporosis about 1 month before the rash appeared.
Al-Attar et al. (2019) [48]	Case report	A 76-year-old female with DRESS syndrome following 10 days after 2nd denosumab administration.

**Table 6:** Summary of osteonecrosis of the jaw (ONJ).

Author	Research	Results/Conclusion
Ferrari et al. (2020) [33]	RCT	Incidence of ONJ: a total of 35 per 100,000 subject-years in the long-term denosumab group. 2. Skeletal Benefit/Risk Ratio: (1) A total of 40 clinical fractures prevented per ONJ case. (2) Considering the severity of adverse events, the benefit/risk ratio is even more favorable than previously estimated.
Watts et al. (2019) [49]	RCT	Findings on ONJ Incidence: (1) OPEs were common in denosumab-treated women. (2) OPEs were associated with an increased ONJ risk, but overall ONJ incidence remained low. 2. ONJ Case Outcomes: All ONJ cases with complete follow-up resolved with treatment.
Beth-Tasdogan et al. (2022) [50]	Review	Non-surgical interventions: Teriparatide; Pentoxifylline and α-tocopherol; Ozone Therapy (OT); Hyperbaric Oxygen Therapy (HBO); Low-Level Laser Therapy (LLLT); Platelet-Derived Growth Factors (PRP, PRGF); Recombinant Bone Morphogenetic Protein (rhBMP). 2. Surgical interventions: Conservative approach; Sequestrectomy and surgical debridement; Aggressive approach; Resection of affected bone followed by reconstruction.

**Table 7:** Summary of denosumab versus bisphosphonates in the treatment of osteoporosis.

Author	Research	Results/Conclusion
Lyu et al. (2019) [51]	Meta-analysis	Denosumab showed significantly greater BMD improvement than bisphosphonates in the lumbar spine, total hip, and femoral neck at 12 and 24 months.
Brown et al. (2021) [52]	Retrospective cohort study	Denosumab improved bone density and reduced fracture risk more effectively than bisphosphonates, and the benefits were observed even in frail and elderly individuals.
Kim et al. (2022) [53]	Retrospective cohort study	Denosumab was superior to zoledronic acid in bone mineral density improvement in frail and elderly patients. Denosumab had a significantly higher treatment persistence rate.
Kobayashi et al. (2024) [54]	Review	Denosumab was associated with lower withdrawal rates due to adverse events and fewer vertebral fractures compared to bisphosphonates, making it a more suitable option for elderly osteoporosis patients.
Sølling (2022) [39]	Review	Bisphosphonates maintain bone quality for years after discontinuation. Denosumab loses its effects rapidly after discontinuation. Rebound effects of denosumab discontinuation include rapid bone mineral density loss and increased fracture risk, especially vertebral fractures.

**Table 8:** Summary of denosumab and improvement of daily function, muscle performance, and quality of life.

Author	Research	Results/Conclusion
Bonnet et al. (2023) [19]	Prospective cohort study	Compared to the bisphosphonate and untreated groups, only the denosumab group showed an increase in ALM and handgrip strength. In denosumab group, changes in ALM and handgrip strength were strongly correlated with lumbar spine BMD changes, whereas no such correlation was observed in the bisphosphonate or untreated groups.
Miedany et al. (2021) [20]	Prospective cohort study	The denosumab group showed a highly significant reduction in fall risk score, along with significant improvements in grip strength, TUG test performance, and gait speed. The zoledronate and alendronate groups demonstrated significant improvements in TUG test and gait speed, respectively, but no significant reduction in fall risk.
Rupp et al. (2022) [21]	Retrospective cohort study	The denosumab group exhibited a significantly greater improvement in chair rising test force than the bisphosphonate group.
Phu et al. (2019) [22]	Prospective cohort study	Denosumab enhanced walking speed, TUG, and FSST performance while reducing the fear of falling, with a tendency toward improvement in SPPB scores and stability limits. Zoledronic acid also improved walking speed and TUG performance, though its effects were less significant than those of denosumab.
Bandeira et al. (2022) [36]	Review	BMD is commonly used to assess bone health and fracture risk, but many people with fractures have osteopenia or normal BMD, which highlights the importance of other factors, such as bone microarchitecture, in determining bone quality. Transiliac Bone Biopsy: Provides direct evaluation of bone microarchitecture but is invasive and not commonly used. HR-pQCT: Offers detailed insights into bone microstructure (volumetric bone density, cortical, and trabecular bone architecture) but not widely available in clinical practice. TBS: (1) A software-based enhancement of lumbar spine DXA. (2) Analyzes texture of lumbar spine DXA images to estimate bone microarchitecture and more accessible alternative to bone biopsy and HR-pQCT.
Shevroja et al. (2023) [55]	Review	TBS can predict fragility fractures independently of BMD and other clinical risk factors. TBS is useful for individuals with FRAX or BMD T-scores near the treatment intervention threshold. TBS, alongside BMD, helps assess response to long-term denosumab treatment (especially over 5+ years). TBS provides additional information on bone microarchitecture when used with BMD. A low TBS may indicate the need for treatments, as it reflects bone microarchitecture degradation.
Brown et al. (2022) [56]	Review	Bone turnover biomarkers (BTBs) in clinical use: Diagnosis of Postmenopausal Osteoporosis: BTBs help detect underlying conditions but are not used for diagnosis. Prediction of Bone Loss in Untreated Postmenopausal Women: BTBs rise due to estrogen deficiency during menopause but are not reliable for predicting individual bone loss. Prediction of Fractures in Untreated Postmenopausal Women: BTBs are not included in standard fracture risk tools like FRAX due to insufficient data. Selection of Pharmacological Treatment: High BTBs suggest antiresorptive therapy, while low BTBs may favor anabolic treatment, but they are not primary factors in treatment selection. Monitoring Response to Therapy in Postmenopausal Osteoporosis: BTBs can assess early response to treatment but are not reliable for predicting long-term fracture risk. Use of BTBs for Optimizing Adherence to Therapy: BTBs could check treatment adherence, but their impact on long-term compliance is unclear. Managing Drug Holidays: Bisphosphonates remain effective after discontinuation, and BTBs can monitor when therapy should resume, though they do not predict future fractures. Managing Cessation of Denosumab Therapy: Denosumab discontinuation raises fracture risk; switching to bisphosphonates may help reduce this risk. Risk Assessment for AFF and ONJ: BTBs may help assess bone metabolism changes, but they do not predict AFF or ONJ.

**Table 9:** Summary of denosumab and Type II DM incidence.

Author	Research	Results/Conclusion
Lyu et al. (2023) [57]	Population-based cohort study	The use of denosumab was linked to a lower risk of developing Type 2 diabetes compared to the use of oral bisphosphonates in osteoporotic adults.
Huang et al. (2024) [5]	Nationwide propensity scored-matched cohort study	Denosumab treatment was associated with a lower risk of incident diabetes.
Henney et al. (2024) [6]	Retrospective analysis	Denosumab treatment is linked to a lower relative risk (RR) of developing Type 2 diabetes, as well as a reduced RR of all-cause mortality and microvascular complications.

**Table 10:** Summary of denosumab and osteoarthritis.

Author	Research	Results/Conclusion
Herrero-Beaumont et al. (2020) [58]	Review	Denosumab may impact the quality of subchondral bone in patients with osteoarthritis by acting as a bone-forming agent.
Shangguan et al. (2024) [59]	Experimental Study	Denosumab may reduce osteoclast activity and prevent chondrocyte apoptosis, potentially alleviating subchondral bone remodeling and cartilage degeneration associated with osteoarthritis.
Yu et al. (2024) [60]	Population-based cohort study	The findings suggest that denosumab may improve subchondral health, potentially slowing the progression of osteoarthritis and delaying the need for total knee arthroplasty.
Wittoek et al. (2024) [61]	RCT	Denosumab may influence the structural damage associated with erosive hand osteoarthritis.

## Data Availability

No datasets were generated or analyzed during the current study.

## References

[1] Udagawa N., Koide M., Nakamura M., Nakamichi Y., Yamashita T., Uehara S., Kobayashi Y., Furuya Y., Yasuda H., Fukuda C. (2021). Osteoclast differentiation by RANKL and OPG signaling pathways. J. Bone Miner. Metab..

[2] Carrillo-López N., Martínez-Arias L., Fernández-Villabrille S., Ruiz-Torres M.P., Dusso A., Cannata-Andía J.B., Naves-Díaz M., Panizo S., European Renal Osteodystrophy (EUROD) Workgroup (2021). Role of the RANK/RANKL/OPG and Wnt/β-Catenin Systems in CKD Bone and Cardiovascular Disorders. Calcif. Tissue Int..

[3] Streicher C., Heyny A., Andrukhova O., Haigl B., Slavic S., Schüler C., Kollmann K., Kantner I., Sexl V., Kleiter M. (2017). Estrogen Regulates Bone Turnover by Targeting RANKL Expression in Bone Lining Cells. Sci. Rep..

[4] Marcadet L., Bouredji Z., Argaw A., Frenette J. (2022). The Roles of RANK/RANKL/OPG in Cardiac, Skeletal, and Smooth Muscles in Health and Disease. Front. Cell Dev. Biol..

[5] Huang H.K., Chuang A.T., Liao T.C., Shao S.C., Liu P.P., Tu Y.K., Lai E.C. (2024). Denosumab and the Risk of Diabetes in Patients Treated for Osteoporosis. JAMA Netw. Open.

[6] Henney A.E., Riley D.R., O’Connor B., Hydes T.J., Anson M., Zhao S.S., Alam U., Cuthbertson D.J. (2024). Denosumab, for osteoporosis, reduces the incidence of type 2 diabetes, risk of foot ulceration and all-cause mortality in adults, compared with bisphosphonates: An analysis of real-world, cohort data, with a systematic review and meta-analysis. Diabetes Obes. Metab..

[7] Kiechl S., Wittmann J., Giaccari A., Knoflach M., Willeit P., Bozec A., Moschen A.R., Muscogiuri G., Sorice G.P., Kireva T. (2013). Blockade of receptor activator of nuclear factor-κB (RANKL) signaling improves hepatic insulin resistance and prevents development of diabetes mellitus. Nat. Med..

[8] Loh K.M., Chen A., Koh P.W., Deng T.Z., Sinha R., Tsai J.M., Barkal A.A., Shen K.Y., Jain R., Morganti R.M. (2016). Mapping the Pairwise Choices Leading from Pluripotency to Human Bone, Heart, and Other Mesoderm Cell Types. Cell.

[9] Hamrick M.W. (2003). Increased bone mineral density in the femora of GDF8 knockout mice. Anat. Rec. Part A Discov. Mol. Cell Evol. Biol..

[10] Pedersen B.K., Febbraio M.A. (2012). Muscles, exercise and obesity: Skeletal muscle as a secretory organ. Nat. Rev. Endocrinol..

[11] Herrmann M., Engelke K., Ebert R., Müller-Deubert S., Rudert M., Ziouti F., Jundt F., Felsenberg D., Jakob F. (2020). Interactions between Muscle and Bone—Where Physics Meets Biology. Biomolecules.

[12] Bonewald L. (2019). Use it or lose it to age: A review of bone and muscle communication. Bone.

[13] Kirk B., Al Saedi A., Duque G. (2019). Osteosarcopenia: A case of geroscience. Aging Med..

[14] Di Monaco M., Castiglioni C., Bardesono F., Milano E., Massazza G. (2020). Sarcopenia, osteoporosis and the burden of prevalent vertebral fractures: A cross-sectional study of 350 women with hip fracture. Eur. J. Phys. Rehabil. Med..

[15] Baud’huin M., Duplomb L., Teletchea S., Lamoureux F., Ruiz-Velasco C., Maillasson M., Redini F., Heymann M.F., Heymann D. (2013). Osteoprotegerin: Multiple partners for multiple functions. Cytokine Growth Factor Rev..

[16] Dufresne S.S., Dumont N.A., Boulanger-Piette A., Fajardo V.A., Gamu D., Kake-Guena S.A., David R.O., Bouchard P., Lavergne É., Penninger J.M. (2016). Muscle RANK is a key regulator of Ca^2+^ storage, SERCA activity, and function of fast-twitch skeletal muscles. Am. J. Physiol. Cell Physiol..

[17] Dufresne S.S., Boulanger-Piette A., Bossé S., Argaw A., Hamoudi D., Marcadet L., Gamu D., Fajardo V.A., Yagita H., Penninger J.M. (2018). Genetic deletion of muscle RANK or selective inhibition of RANKL is not as effective as full-length OPG-fc in mitigating muscular dystrophy. Acta Neuropathol. Commun..

[18] Cummings S.R., San Martin J., McClung M.R., Siris E.S., Eastell R., Reid I.R., Delmas P., Zoog H.B., Austin M., Wang A. (2009). Denosumab for prevention of fractures in postmenopausal women with osteoporosis. N. Engl. J. Med..

[19] Bonnet N., Bourgoin L., Biver E., Douni E., Ferrari S. (2023). RANKL inhibition improves muscle strength and insulin sensitivity and restores bone mass. J. Clin. Investig..

[20] Miedany Y.E., Gaafary M.E., Toth M., Hegazi M.O., Aroussy N.E., Hassan W., Almedany S., Nasr A., Bahlas S., Galal S. (2021). Is there a potential dual effect of denosumab for treatment of osteoporosis and sarcopenia?. Clin. Rheumatol..

[21] Rupp T., von Vopelius E., Strahl A., Oheim R., Barvencik F., Amling M., Rolvien T. (2022). Beneficial effects of denosumab on muscle performance in patients with low BMD: A retrospective, propensity score-matched study. Osteoporos. Int..

[22] Phu S., Bani Hassan E., Vogrin S., Kirk B., Duque G. (2019). Effect of Denosumab on Falls, Muscle Strength, and Function in Community-Dwelling Older Adults. J. Am. Geriatr. Soc..

[23] Yu C., Du Y., Peng Z., Ma C., Fang J., Ma L., Chen F., Zhang C., Geng R., Zhang Y. (2023). Research advances in crosstalk between muscle and bone in osteosarcopenia (Review). Exp. Ther. Med..

[24] Corallini F., Rimondi E., Secchiero P. (2008). TRAIL and osteoprotegerin: A role in endothelial physiopathology?. Front. Biosci..

[25] Yano K., Tsuda E., Washida N., Kobayashi F., Goto M., Harada A., Ikeda K., Higashio K., Yamada Y. (1999). Immunological characterization of circulating osteoprotegerin/osteoclastogenesis inhibitory factor: Increased serum concentrations in postmenopausal women with osteoporosis. J. Bone Miner. Res..

[26] Price P.A., June H.H., Buckley J.R., Williamson M.K. (2001). Osteoprotegerin inhibits artery calcification induced by warfarin and by vitamin D. Arterioscler. Thromb. Vasc. Biol..

[27] Wang F., Li W., Gao C., Zhou B., Ma M. (2008). Osteoprotegerin/RANK/RANKL axis in cardiac remodeling due to immuno-inflammatory myocardial disease. Exp. Mol. Pathol..

[28] Kawakami R., Nakagami H., Noma T., Ohmori K., Kohno M., Morishita R. (2016). RANKL system in vascular and valve calcification with aging. Inflamm. Regen..

[29] Davenport C., Harper E., Forde H., Rochfort K.D., Murphy R.P., Smith D., Cummins P.M. (2016). RANKL promotes osteoblastic activity in vascular smooth muscle cells by upregulating endothelial BMP-2 release. Int. J. Biochem. Cell Biol..

[30] Davenport C., Harper E., Rochfort K.D., Forde H., Smith D., Cummins P.M. (2018). RANKL Inhibits the Production of Osteoprotegerin from Smooth Muscle Cells under Basal Conditions and following Exposure to Cyclic Strain. J. Vasc. Res..

[31] Rochette L., Meloux A., Rigal E., Zeller M., Cottin Y., Vergely C. (2019). The Role of Osteoprotegerin and Its Ligands in Vascular Function. Int. J. Mol. Sci..

[32] Ferrari S., Butler P.W., Kendler D.L., Miller P.D., Roux C., Wang A.T., Huang S., Wagman R.B., Lewiecki E.M. (2019). Further Nonvertebral Fracture Reduction Beyond 3 Years for Up to 10 Years of Denosumab Treatment. J. Clin. Endocrinol. Metab..

[33] Ferrari S., Lewiecki E.M., Butler P.W., Kendler D.L., Napoli N., Huang S., Crittenden D.B., Pannacciulli N., Siris E., Binkley N. (2020). Favorable skeletal benefit/risk of long-term denosumab therapy: A virtual-twin analysis of fractures prevented relative to skeletal safety events observed. Bone.

[34] Kendler D.L., Chines A., Brandi M.L., Papapoulos S., Lewiecki E.M., Reginster J.Y., Muñoz Torres M., Wang A., Bone H.G. (2019). The risk of subsequent osteoporotic fractures is decreased in subjects experiencing fracture while on denosumab: Results from the FREEDOM and FREEDOM Extension studies. Osteoporos. Int..

[35] Kendler D.L., Cosman F., Stad R.K., Ferrari S. (2022). Denosumab in the Treatment of Osteoporosis: 10 Years Later: A Narrative Review. Adv. Ther..

[36] Bandeira F., de Oliveira L.B., Bilezikian J.P. (2022). Long-term consequences of osteoporosis therapy with denosumab. Arch. Endocrinol. Metab..

[37] Di Lorenzo L. (2023). Denosumab in elderly osteoporotic patients. A narrative review. Clin. Ter..

[38] Cummings S.R., Ferrari S., Eastell R., Gilchrist N., Jensen J.B., McClung M., Roux C., Törring O., Valter I., Wang A.T. (2018). Vertebral Fractures After Discontinuation of Denosumab: A Post Hoc Analysis of the Randomized Placebo-Controlled FREEDOM Trial and Its Extension. J. Bone Miner. Res..

[39] Sølling A.S., Tsourdi E., Harsløf T., Langdahl B.L. (2023). Denosumab Discontinuation. Curr. Osteoporos. Rep..

[40] Bridgeman M.B., Pathak R. (2011). Denosumab for the reduction of bone loss in postmenopausal osteoporosis: A review. Clin. Ther..

[41] Tsvetov G., Amitai O., Shochat T., Shimon I., Akirov A., Diker-Cohen T. (2020). Denosumab-induced hypocalcemia in patients with osteoporosis: Can you know who will get low?. Osteoporos. Int..

[42] Dadana S., Gundepalli S., Kondapalli A. (2023). Severe Refractory Hypocalcemia Caused by Denosumab. Cureus.

[43] Bird S.T., Smith E.R., Gelperin K., Jung T.H., Thompson A., Kambhampati R., Lyu H., Zhao H., Zhao Y., Zhu Y. (2024). Severe Hypocalcemia with Denosumab Among Older Female Dialysis-Dependent Patients. JAMA.

[44] Kunizawa K., Hiramatsu R., Hoshino J., Mizuno H., Ozawa Y., Sekine A., Kawada M., Sumida K., Hasegawa E., Yamanouchi M. (2020). Denosumab for dialysis patients with osteoporosis: A cohort study. Sci. Rep..

[45] Diker-Cohen T., Rosenberg D., Avni T., Shepshelovich D., Tsvetov G., Gafter-Gvili A. (2020). Risk for Infections During Treatment with Denosumab for Osteoporosis: A Systematic Review and Meta-analysis. J. Clin. Endocrinol. Metab..

[46] Huang S.T., Chiu T.F., Chiu C.W., Kao Y.N., Wang I.K., Chang C.T., Li C.Y., Sun C.S., Lin C.L., Yu T.M. (2023). Denosumab treatment and infection risks in patients with osteoporosis: Propensity score matching analysis of a national-wide population-based cohort study. Front. Endocrinol..

[47] King B.J., Lehman J.S., Sartori Valinotti J.C. (2018). Denosumab-induced cutaneous hypersensitivity reaction with distinct clinical and histopathologic findings. J. Cutan. Pathol..

[48] Al-Attar M., De Santis M., Massarotti M. (2019). DRESS syndrome in response to Denosumab: First documented case report. Bone Rep..

[49] Watts N.B., Grbic J.T., Binkley N., Papapoulos S., Butler P.W., Yin X., Tierney A., Wagman R.B., McClung M. (2019). Invasive Oral Procedures and Events in Postmenopausal Women with Osteoporosis Treated with Denosumab for Up to 10 Years. J. Clin. Endocrinol. Metab..

[50] Beth-Tasdogan N.H., Mayer B., Hussein H., Zolk O., Peter J.U. (2022). Interventions for managing medication-related osteonecrosis of the jaw. Cochrane Database Syst. Rev..

[51] Lyu H., Jundi B., Xu C., Tedeschi S.K., Yoshida K., Zhao S., Nigwekar S.U., Leder B.Z., Solomon D.H. (2019). Comparison of Denosumab and Bisphosphonates in Patients with Osteoporosis: A Meta-Analysis of Randomized Controlled Trials. J. Clin. Endocrinol. Metab..

[52] Brown J.P., Adachi J.D., Schemitsch E., Tarride J.E., Brown V., Bell A., Reiner M., Oliveira T., Motsepe-Ditshego P., Burke N. (2021). Mortality in older adults following a fragility fracture: Real-world retrospective matched-cohort study in Ontario. BMC Musculoskelet. Disord..

[53] Kim S.J., Kim J.W., Lee D.W. (2022). Denosumab versus zoledronic acid in elderly patients after hip fracture. J. Orthop. Surg..

[54] Kobayashi T., Morimoto T., Ito K., Mawatari M., Shimazaki T. (2024). Denosumab vs. bisphosphonates in primary osteoporosis: A meta-analysis of comparative safety in randomized controlled trials. Osteoporos. Int..

[55] Shevroja E., Reginster J.Y., Lamy O., Al-Daghri N., Chandran M., Demoux-Baiada A.L., Kohlmeier L., Lecart M.P., Messina D., Camargos B.M. (2023). Update on the clinical use of trabecular bone score (TBS) in the management of osteoporosis: Results of an expert group meeting organized by the European Society for Clinical and Economic Aspects of Osteoporosis, Osteoarthritis and Musculoskeletal Diseases (ESCEO), and the International Osteoporosis Foundation (IOF) under the auspices of WHO Collaborating Center for Epidemiology of Musculoskeletal Health and Aging. Osteoporos. Int..

[56] Brown J.P., Don-Wauchope A., Douville P., Albert C., Vasikaran S.D. (2022). Current use of bone turnover markers in the management of osteoporosis. Clin. Biochem..

[57] Lyu H., Zhao S.S., Zhang L., Wei J., Li X., Li H., Liu Y., Yin P., Norvang V., Yoshida K. (2023). Denosumab and incidence of type 2 diabetes among adults with osteoporosis: Population based cohort study. BMJ.

[58] Herrero-Beaumont G., Roman-Blas J.A., Mediero A., Sánchez-Pernaute O., Largo R. (2020). Treating osteoporotic osteoarthritis, or the art of cutting a balding man’s hair. Osteoarthr. Cartil..

[59] Shangguan L., Ding M., Wang Y., Xu H., Liao B. (2024). Denosumab ameliorates osteoarthritis by protecting cartilage against degradation and modulating subchondral bone remodeling. Regen. Ther..

[60] Yu T.C., Wu W.T., Lee R.P., Chen I.H., Wang J.H., Wen S.H., Yeh K.T. (2024). Incidence of Total Knee Arthroplasty in Older Females with Knee Osteoarthritis and Osteoporosis Treated with Denosumab Compared with Those Treated Using Bisphosphonates: A Population-Based Cohort Study. Life.

[61] Wittoek R., Verbruggen G., Vanhaverbeke T., Colman R., Elewaut D. (2024). RANKL blockade for erosive hand osteoarthritis: A randomized placebo-controlled phase 2a trial. Nat. Med..

[62] Cai D., Yuan M., Frantz D.F., Melendez P.A., Hansen L., Lee J., Shoelson S.E. (2005). Local and systemic insulin resistance resulting from hepatic activation of IKK-beta and NF-κB. Nat. Med..

[63] Kondegowda N.G., Fenutria R., Pollack I.R., Orthofer M., Garcia-Ocaña A., Penninger J.M., Vasavada R.C. (2015). Osteoprotegerin and Denosumab Stimulate Human Beta Cell Proliferation through Inhibition of the Receptor Activator of NF-κB Ligand Pathway. Cell Metab..

